# Selective and non‐selective bottlenecks as drivers of the evolution of hypermutable bacterial loci

**DOI:** 10.1111/mmi.14453

**Published:** 2020-03-17

**Authors:** Megan De Ste Croix, Jonathan Holmes, Joseph J. Wanford, E. Richard Moxon, Marco R. Oggioni, Christopher D. Bayliss

**Affiliations:** ^1^ Department of Genetics and Genome Biology University of Leicester Leicester UK; ^2^ Department of Paediatrics University of Oxford Medical Sciences Division John Radcliffe Hospital Oxford UK

**Keywords:** antigenic variation, bottlenecks, hypermutable, mutation rates, phase variation, selection, localised hypermutation

## Abstract

Bottlenecks reduce the size of the gene pool within populations of all life forms with implications for their subsequent survival. Here, we examine the effects of bottlenecks on bacterial commensal‐pathogens during transmission between, and dissemination within, hosts. By reducing genetic diversity, bottlenecks may alter individual or population‐wide adaptive potential. A diverse range of hypermutable mechanisms have evolved in infectious agents that allow for rapid generation of genetic diversity in specific genomic loci as opposed to the variability arising from increased genome‐wide mutation rates. These localised hypermutable mechanisms include multi‐gene phase variation (PV) of outer membrane components, multi‐allele PV of restriction systems and recombination‐driven antigenic variation. We review selected experimental and theoretical (mathematical) models pertaining to the hypothesis that localised hypermutation (LH) compensates for fitness losses caused by bottlenecks and discuss whether bottlenecks have driven the evolution of hypermutable loci.

## Graphical abstract

Bottlenecks produce dramatic reductions in the genetic diversity of bacterial populations. Bottleneck events can be selective or non‐selective and occur frequently, particularly during transmission between hosts. Multiple mutational strategies have evolved in bacteria that can regenerate genetic diversity within surviving populations following a major reduction in population size.

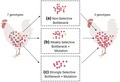

## 1 INTRODUCTION TO THE IMPACT OF BOTTLENECKS ON BACTERIAL GENETIC DIVERSITY

**Figure 1:**
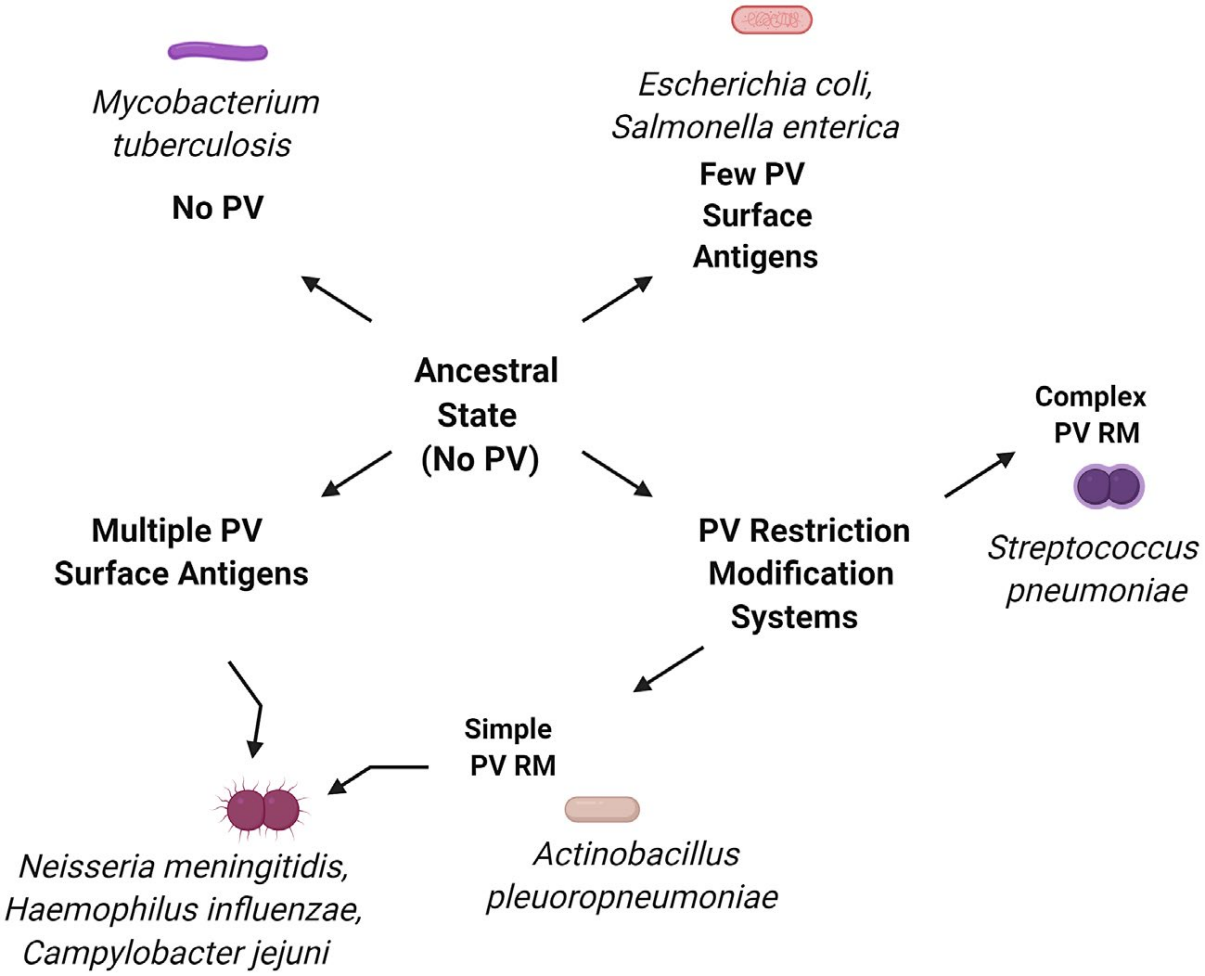
Overview of the distribution of phase‐variable genes. This figure depicts the putative evolution of PV in prokaryotes from an ancestral organism lacking PV to a diverse range of PV patterns that differ in the number (few or multiple), function (surface antigen and restriction‐modification, RM) and complexity (simple or complex) of the phase‐variable loci. Exemplar species are provided for each pattern. Image created with BioRender

**Figure 2:**
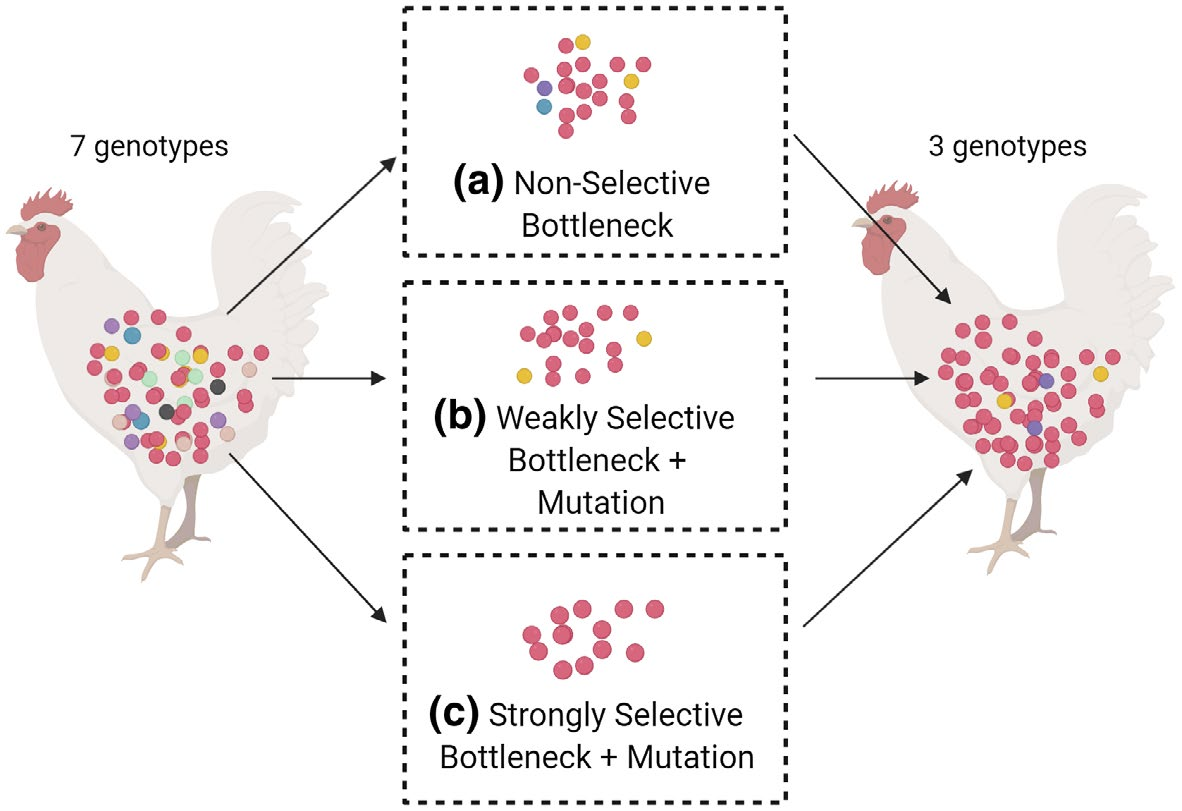
Impact of selective and non‐selective bottlenecks on the genetic diversity of bacterial populations during transmission between hosts. This figure depicts transmission of a bacterial species from one host to another by the faecal‐oral route with an initial step of excretion from a large genetically diverse population, consisting of seven genotypes, of a small subset of the population into the environment followed by a subsequent step of acquisition by and colonisation of a second host by a less diverse population, consisting of three genotypes. The genotypes represent different combinations of alleles or phase variants of multiple genes. (a) Non‐selective bottlenecks. In this mode, there is random inclusion of particular genotypes from the starting population into the transmitted population during both the initial excretion and secondary acquisition steps. These bottlenecks strongly skew the population structure without any selection such that dominant genotypes occur by chance with no guarantee of the same pattern being observed during another similar transmission event. (b) Mixed bottlenecks. In this model, the purple, yellow and blue variants are weakly adaptive for survival in the environment. However, due to the non‐selective excretion bottleneck, only the adaptive yellow and non‐adaptive red variants pass through the initial bottleneck and colonise the second host. (c) Selective bottlenecks. In this case, the red variant type has a much higher fitness for survival in the environment than other variants. Hence only this variant is transmitted. In this selective bottleneck, selection of the red variant produces a significant reduction in population diversity of the transmitted population that would be reproducible during another similar transmission event. High mutation rates allow for recovery of some additional genetic variation during colonisation of the second host in both B and C. Image created with BioRender

The evolution and spread of infectious disease agents are underpinned by an ability to generate high levels of genetic variation enabling rapid acquirement of novel adaptive traits. A key on‐going paradigm shift in our understanding of genetic variability in pathogenic bacteria was the demonstration that localised hypermutation (LH) due to hypermutable DNA elements was responsible for the phase variation (PV) phenomenon of rapid ON and OFF switches in surface antigens and restriction‐modification systems (RM). We now recognise that LH and PV are widely distributed across bacterial pathogens and commensals but with significant diversity in the numbers of loci and in the types of functions encoded by these loci (Figure 1). The importance of genetic variability to infection has been recognised by careful epidemiological investigations whose ability to detect genetic determinants of disease has been enhanced by the genomics revolution. An under‐appreciated conundrum of the infectious disease lifestyle is that genetic diversity is severely impacted by bottlenecks during transmission between and within‐hosts (Figure 2). The following sections review our current understanding of the nature of bottlenecks during bacterial infections and how bottlenecks impact on the genetic diversity. We consider the diversity of LH mechanisms and the use of mathematical models, and simulations to dissect and explore the interplay between bottlenecks and LH‐driven genetic diversification.

## 2 SELECTIVE AND NON‐SELECTIVE BOTTLENECKS INFLUENCE BACTERIAL COMMENSAL‐PATHOGEN GENETIC DIVERSITY DURING SPREAD WITHIN AND TRANSMISSION BETWEEN HOSTS

Bottlenecks are a short‐hand to describe the phenomenon of a rapid reduction in the number of organisms in a population leading to loss of genetic variants (Figure 2). Conceptually, two opposing types of bottlenecks can be defined, selective and non‐selective. While these differing types of bottlenecks may occur in isolation and have defining features, biological situations are often complex and these two types of bottlenecks may occur simultaneously or, due to varying strengths of selection, overlap. Selective bottlenecks occur when selection acts on one or a subset of genetically encoded phenotypes in a population. In this case, the degree of bottlenecking is directly linked to the strength of selection with differences in the fitness advantages of the genetic variants for surviving the bottleneck altering the genetic structure of the whole surviving population. Non‐selective bottlenecks occur when a population is reduced in size, sometimes to only a few cells, through chance, non‐selective events such as a physical reduction in the population during transmission to a new host. In non‐selective bottlenecks, the effect on the genetic variants within the surviving population is random. This effect may be observed as each biological replicate of the same experiment having a different population structure and hence high genetic divergence from the starting population and between biological replicates (see Aidley, Rajopadhye, Akinyemi, Lango‐Scholey & Bayliss, 2017). Contrastingly, selective bottlenecks, particularly when selection is strong, may lead to high divergence from the starting population but low divergence between biological replicates of the output populations due to selection of a common genotype. Thus selective and non‐selective bottlenecks create changes in population structure that can be extreme when a single cell bottleneck occurs.

### 2.1 Experimental examples of non‐selective bottlenecks

Early evidence of infection‐associated bottlenecks came from studies of pneumococcal pathogenesis and the observation that intravenous injection was followed by a major reduction in population size prior to establishment of high‐level bacteraemia (Wright, 1927). Gerlini and Colleagues (2014) demonstrated that this bottleneck can be extremely narrow (10–100 cells), and in some cases can even result in clonal expansion from a single surviving bacterium. Very recently, Ercoli and Colleagues (2018) utilised microscopy to connect this systemic bottleneck with permissiveness for pneumococcal replication in a subset of CD169+ splenic macrophages. Infection and bacterial replication within these cells was essential for disease, as evidenced by survival of mice treated with an anti‐CD169 monoclonal antibody, however, infection of these cells did not impose selection for a particular genotype but was instead stochastic for isogenic genotypes. In parallel experiments, Zafar, Kono, Wang, Zangari and Weiser, (2016) developed an infection model for studying transmission bottlenecks wherein pneumococcal shedding from the nasopharynx of infected mice leads to transmission to non‐infected littermates. Using a mixed bacterial population with differing antibiotic resistance profiles, the authors observed that there is a narrow population bottleneck during pneumococcal transmission to a new host but not during exit from the nasal cavity (Kono et al., 2016). In both cases, re‐inoculation of output organisms conferred no advantage in subsequent experiments providing evidence of a non‐selective bottleneck.

### 2.2 Experimental example of within‐host, selective bottlenecks

Bayliss and colleagues (2008) utilised bacterial escape, from killing by a bactericidal antibody, mediated by PV to demonstrate how immune competent hosts could impose a selective bottleneck. Exposure of *Neisseria meningitidis* to a bactericidal monoclonal antibody specific for a phase‐variable LOS epitope (Banerjee et al., 1998) resulted in selection for OFF variants as the ON variants were killed in an antibody‐dependent manner both in vitro and in a passive immunisation experiment in infant rats. Survival of the bacterial population was dependent on the relative amounts of bacterial cells and antibody and the rate of ON‐OFF switching. These experiments are an example of a strong within‐host bottleneck that selects between isogenic phase variants resulting in survival of the variants that lack expression of a specific surface‐exposed immune target.

### 2.3 Selective bottlenecks in systemic dissemination of *Salmonella* in the face of vaccine‐induced adaptive immunity and antibiotic selection

Using *Salmonella* as a model organism, Meynell (1957) first proposed the theory of independent action to explain the predominantly clonal bacteraemia arising from mixed oral infection with *Salmonella typhimurium.* This theory proposes that each individual (e.g. bacterial cell) in a population has an independent probability to initiate an infection without any cooperative or synergistic interactions from other individuals in the population so that infections become progressively more likely to occur for every increase in the infecting population size.

Myriad studies with populations containing mixtures of differentially tagged, but otherwise isogenic, *Salmonella* cells have inferred the presence of multiple within‐host bottlenecks. Han Lim and Colleagues (2014) detected multiple independent bottlenecks during both colonisation of the murine gut and dissemination to systemic compartments. Infection of vaccinated mice revealed that bacterial populations in the systemic compartment were entirely uncoupled from those in the Peyer's patch, indicating a critical role for adaptive immunity in the imposition of bottlenecks during systemic disease.

**Figure 3:**
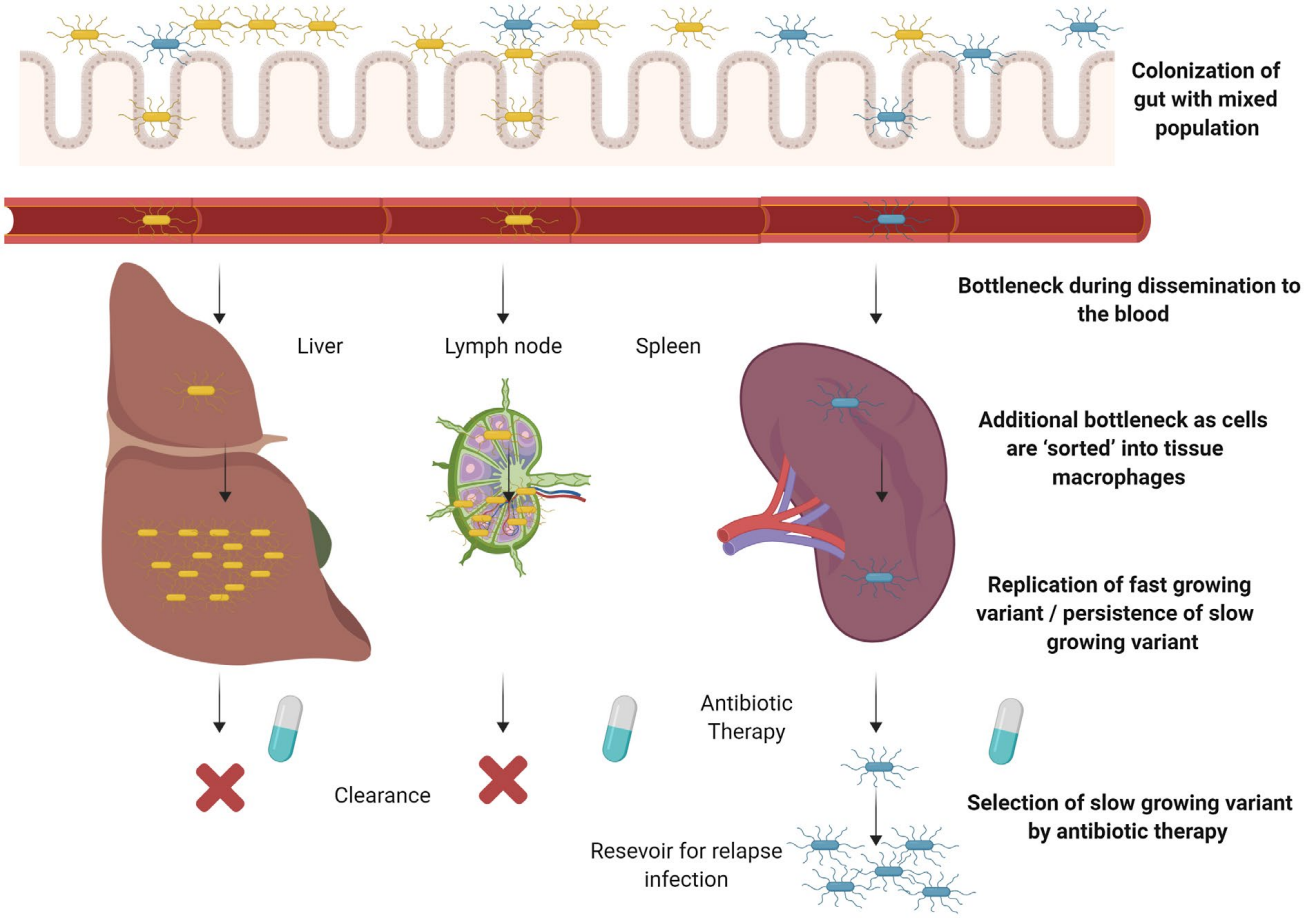
Spread of a bacterial pathogen within a host is subject to multiple selective and non‐selective bottlenecks that impact on disease outcome. A phase‐variable bacterial species is depicted that exists in two phase variation states (blue and yellow) in the gastrointestinal tract of a host. This organism penetrates the mucosal lining of the gut and replicates in the bloodstream and subsequently spreads to internal organs. The yellow variants are able to replicate at fast rates in internal organs but are rapidly cleared by immune effector cells and/or antibiotic treatment. In contrast, the blue variants invade hosts in the organs where they replicate slowly and are able to resist antibiotic clearance due to poor intracellular penetration of antibiotics and slow growth rates. These organisms provide a reservoir for relapse of an infection due to the generation of antibiotic resistance or cessation of treatment. Each stage of this process introduces either narrow non‐selective bottlenecks (i.e. dissemination to blood and tissue macrophages) or a stringent selective bottleneck (i.e. survival of antibiotic therapy) such that disease only occurs in a subset of hosts following the stochastic effects of sorting between the two variants as they pass through each of the bottlenecks. Image created with BioRender

Bacterial growth rates of within‐host populations are often heterogeneous (Balaban, Merrin, Chait, Kowalik & Leibler, 2004). This is important as several antibiotics are only efficacious against actively growing bacteria, such as ampicillin and ciprofloxacin (Lobritz et al., 2015), and hence can impose a strong growth rate selection (Udekwu, Parrish, Ankomah, Baquero & Levin, 2009; White, Toothaker, Smith & Slattery, 1989). Rossi et al. (Rossi et al., 2017) used a panel of isogenically tagged wild‐type and slow‐growing mutant *Salmonella* to infect mice followed by treatment with either ampicillin or ciprofloxacin (Figure 3). A strong bottleneck was observed during initial colonisation favouring fast‐growing variants. However, antibiotic treatment resulted in a greater reduction in the counts of fast‐growing variants than slow‐growing variants whose lower clearance rates resulted in persistence for >13 days after therapy. The implication of this experiment is that the initial non‐specific infection bottleneck could shuffle fast‐ and slow‐growing variants into different compartments resulting in tissue heterogeneity for clearance in response to antibiotic therapy.

## 3 HYPERMUTABLE AND HYPERVARIABLE LOCI ARE WIDESPREAD IN BACTERIAL COMMENSAL‐PATHOGENS

In 1922, Andrewes identified a novel phenomenon (termed PV); stochastic, reversible, high frequency alterations in the flagella phenotypes of *Salmonella* (Andrewes, 1922). Discovery of further examples of PV led to the unexpected discovery of parallel evolution of multiple mechanisms for generating variation in a diverse range of bacterial species and of the key role of mutability. The latter aspect was codified by Moxon, Rainey, Nowak and Lenski (1994) who proposed the notion of hypermutable genomic regions, referred to as LH, and separated genes into two broad classes – contingency loci, subject to LH, and genes in which mutation is constrained, such as those with ‘core’ metabolic or replicative functions. LH is defined as a region of the genome that has a mutation or recombination rate that is significantly higher than the genome‐wide rate and usually exceeds 10^–5^ mutations/locus/division. This definition is somewhat arbitrary as DNA sequences exhibit a spectrum of mutation and recombination rates and variations in the genome‐wide rates both within and between species. Contingency loci cover LH‐mediated PV or high frequency antigenic variation but the term is often stretched to include methylation‐based mechanisms of PV as exemplified by the *E. coli* P‐pilus. It is also notable that this phenomenon has evolved in eukaryotes as exemplified by somatic hypermutation for generation of antibody diversity.

A key early observation was that the majority of contingency loci encode bacterial surface antigens or indirect modifiers of these antigens (Figure 1). Subsequently, genome sequencing revealed a surprising, but common, observation for ON/OFF switching of Type I and Type III RM systems, termed herein a simple PV RM (Ershova, Rusinov, Spirin, Karyagina, & Alexeevski, 2015; Moxon, Bayliss, & Hood, 2006). The complexity of RM PV, whose changes in expression are potentially self‐lethal, was deepened by the detection of rapid switching between multiple Target Recognition Domains (TRDs) with different recognition sequences (see below), termed herein a complex PV RM.

A final generic observation is that LH is not only widespread but exhibits variations in prevalence between and within species (Figure 1). Some species have only one hypermutable locus while others have multiple. Similarly, some species only have a simple or complex PV RM. The differential species distributions and individual genome patterns hint at a multifaceted evolutionary process with arguments for diverse selective pressures, on‐going selection, rapid genetic sweeps by adaptive loci and rapid/slow loss of non‐adaptive PV mechanisms. We will argue below that bottlenecks are a critical and generally important aspect in the evolutionary pathways leading to the LH phenomenon.

## 4 SPECIFIC HYPERMUTABLE MECHANISMS FOUND IN BACTERIAL PATHOGENS

In order to support the generic view of the previous section, we review some recent findings on distributions of LH and how hypermutable switches contribute to within‐host evolution.

### 4.1 Improving discovery of PV‐associated SSRs

Phasome*It* is a new programme (Aidley, Wanford, Green, Sheppard & Bayliss, 2018) that overcomes limitations of previous simple sequence repeat (SSR) discovery programmes by automating the discovery and linkage of prokaryotic SSR in genome sequences to the potential for the SSR to elicit PV. Application of this programme to multiple *Campylobacter* and *Neisseria* species detected wide variations in SSR‐mediated PV with some species only having 1–5 loci while others have 30–50.

### 4.2 A wide distribution of invertons in genomes

High frequency ON/OFF PV can also be mediated by site‐specific recombinases that generate promoter inversions. Jiang and Colleagues (2019) developed a programme, PhaseFinder, for finding intergenic invertible DNA regions (invertons) in bacterial genomes. Application of PhaseFinder to pre‐existing whole genome seuences (WGS) for ~2,000 genomes revealed 4,686 putative invertons with an over‐representation in host‐associated bacteria. Many intervons were associated with metabolically expensive genes, including mediators of antibiotic resistance, a novel finding for LH. By studying bacterial isolates from children with and without exposure to antibiotics, Jiang et al. identified three antibiotic resistance genes regulated by invertons, including the macrolide resistance gene *ermG*. The increase in long read sequencing data is facilitating detection of invertons by PhaseFinder as this program relies on the presence of both inverton orientations in the sequence data (Jiang et al., 2019).

### 4.3 Simple PV RM and complex PV RM systems are widespread

Atack and colleagues (2018) examined 393 *Streptococcis suis* genomes and found 303 simple PV RM systems. This finding emphasises the ubiquity of these loci and the likelihood of strong selection for loci capable of stochastic variations in RM expression. Kwun, Oggioni, Ste Croix, Bentley and Croucher (2018) demonstrated that the pneumococcal SpnIV or *tvr* locus mediates PV between enzymes capable of recognising nine different DNA sequences. Typically, Type I RM systems contain three genes; *hsdR,* encoding a DNA endonuclease; *hsdM, *encoding a DNA methylase; and *hsdS,* encoding a protein with two TRDs and responsible for DNA recognition by both the methylase and endonuclease. In some systems, such as that described by Kwun et al., reversible switches between *hsdS* TRDs occur at high rates. Switching is mediated by direct repeats flanking each TRD and permits the generation of multiple different *hsdS* TRD combinations. Each TRD combination is capable of recognising, methylating and restricting a different DNA sequence. These differing SpnIV methylation patterns were shown to act as a barrier to the acquisition of large genomic islands between pneumococcal strains. A similar mechanism of TRD exchange was found in Type III *mod* genes of *Helicobacter pylori*. Termed Domain Movement (DoMo), TRD switching is facilitated by conserved regions flanking the TRD sequences (Furuta, Kawai, Uchiyama & Kobayashi, 2011). There are at least three patterns of TRD movement; (a) switching between two orientations in a single operon; (b) interchange of TRDs between different loci; and (c) movement of TRD domains between two positions and two orientations resulting in eight TRD combinations. The latter type provides the greatest capacity for variation in methylation patterns.

### 4.4 Elucidation of the switching mechanisms in complex RM systems

Typical Type I RM systems contain separate genes for *hsdS, hsdR* and *hsdM*. A change between one recognition site and another can occur by acquisition of a new *hsdS* allele following horizontal gene transfer. However, several *hsd* operons have additional, non‐functional, divergent *hsdS* genes. Recent work shows that these non‐functional *hsdS* genes act as a reservoir for high frequency exchange of TRDs between functional and non‐functional genome positions leading to alterations in the methylation target sequence (De Ste Croix, 2017). This TRD exchange is frequent, rapid and reversible, and can be mediated either by a locus‐associated site‐specific recombinase or RecA‐mediated recombination. A key mechanistic feature is that DNA sequences as short as 13–15bp can facilitate rearrangements between genomic positions (De Ste Croix, 2017; Furuta & Kobayashi, 2012; Li, Li, Li, Wang & Zhang, 2019). The outcome of this hyper‐recombination mechanism is rapid switching between multiple, alternative methylation patterns but with differing *hsdS* profiles and switching rates depending on the initial operon orientation.

### 4.5 Integrons and Shufflons as mechanisms for gene memory

Integrons (see review by Escudero, Loot, Nivina & Mazel, 2014) can act as a ‘low‐cost memory store’ for certain type of genes. Integrons consist of a series of promoterless genes, a single promoter and a recombinase. Promoterless genes allow for the presence in a genome of a potentially beneficial gene without the burden of expression. A promoter close to the integration site of these elements permits expression of only the nearest gene while a tyrosine recombinase encoded in the integron facilitates recombination and movement of genes between expressed and non‐expressed positions. The ubiquity of mobile integrons, containing almost exclusively antimicrobial resistance genes, within healthcare settings demonstrates the importance of integrons for adaptive environmental responses. Shufflons are another site‐specific recombination system that can generate diversity in expression of specific genes (Sekizuka et al., 2017). The incompatibility (Inc) plasmids, I1 and I2, contain partial open reading frames for up to four different *pilV* alleles that can be rearranged by the recombinase Rci using a conserved region. Variation in the expressed PilV protein contributes to recognition of recipient cells during plasmid transmission and hence facilitates plasmid exchange within and between a wide range of *Enterobacteriaceae *species (Sekizuka et al., 2017).

### 4.6 Evolution of SSRs during clonal expansion

A key recent finding was that several phase‐variable genes, encoding outer membrane proteins (OMPs), had evolved longer SSRs as compared to the parental clone during expansion and persistence within the UK of a serogroup W cc11 sub‐clone of *N. meningitidis* (Green et al., 2019). Higher repeat numbers are more mutable and increase switching between ON and OFF expression states. This increase in the variability of OMP expression was proposed to have facilitated rapid expansion of this clone by enhancing survival of host immune responses. As these immune responses will vary between hosts, it was proposed that the enhanced variability facilitates the survival of transmission bottlenecks during host‐to‐host spread.

### 4.7 Within‐host evolution of SSRs enhances host persistence

In a study of eight contingency loci, Alamro and Colleagues (2014) found that persistent, asymptomatic carriage of *N. meningitidis* was associated with reductions in the combined expression states of OMP‐encoding PV loci. High levels of serum antibodies to the capsule and one of these OMPs were present in the carriers studied suggesting that antibody‐mediated selection drives these changes. Using WGS of multiple longitudinal non‐typeable *Haemophilus influenzae* (NTHi) isolates from 61 persistently chronic obstructive pulmonary disease (COPD) patients, Pettigrew *et al*. found that increases in repeat number were positively correlated with length of persistence (Pettigrew et al., 2018). A more detailed analysis of closed genome sequences of four isolates revealed 22 SSR‐containing genes, with variations in repeat number of the HMW1A/HMW2A‐related adhesins and the haemoglobin‐haptoglobin‐binding proteins occurring in all four isolates. This within‐host variation may facilitate adaptation to selective bottlenecks as mediated by fluctuations in nutrient availability, innate and adaptive immune responses and shifting adhesive properties of mucosal surfaces.

### 4.8 Advances in understanding the roles of phase‐variable RM systems

SSR‐mediated switches in expression of a *Campylobacter jejuni* simple RM PV system can generate resistance to infection by phages (Anjum et al., 2016). While work in this area is limited, this finding supports the notion that phase‐variable RM systems have evolved for phage defence (Hoskisson & Smith, 2007). Another layer of complexity for PV RMs genes was added when links were detected between switches in RM expression and phenotypic variation as an outcome of differential genome methylation (termed the phasevarion; Srikhanta, Fox, & Jennings, 2010). The mechanistic basis for these links is still being evaluated and there are outstanding questions such as whether phasevarions are adaptive and why is there strain‐to‐strain variation in the TRDs of these phase‐variable RMs if the main function is gene regulation. Nevertheless, there are multiple observations linking ON/OFF switching of the methyltransferase of both simple and complex RM systems with alterations in site‐specific methylation states and differences in genic and phenotypic expression (Atack et al., 2018). For example, sub‐components of the *hsdS* gene of the well‐characterised complex PV RM of *Streptococcus pneumoniae*, SpnIII, can exist in one of six possible orientations. Each of these unique TRD combinations is associated with a different DNA methylation pattern and some of them can impact on invasive disease potential in mice and the invasion‐associated opaque colony phenotype (Li et al., 2019, 2016; Manso et al., 2014; Oliver, Roy, Kumar, Lefkowitz & Swords, 2017). The simplicity of these findings is challenged by variable experimental observations, suggesting that genetic background may influence links between methylation and colony opacity, and by the extensive *hsdS* allelic variation resulting in differing dominant methylation patterns among pneumococcal strains (De Ste Croix et al., 2019). Nevertheless, switches in expression of this complex RM system have the potential to influence disease progression and could have unpredictable interactions with the bottlenecks occurring during within‐host spread of pneumococci as described above.

## 5 APPLICATION OF MATHEMATICAL MODELS TO UNDERSTANDING HOW LH COMPENSATES FOR THE LOSS OF ADAPTIVE POTENTIAL IMPOSED BY A BOTTLENECK

Mathematical models are a key tool of mutational research due to the stochastic nature and population‐based effects of mutational process. Our focus is on modelling the interplay between bottlenecks and LH and on two important questions; ‘Does LH provide an adaptive advantage following a bottleneck?’; and ‘Is the selective advantage of adapting to a bottleneck sufficient to drive LH evolution’. The critical problem for bacterial populations is that bottlenecks often appear rapidly, unpredictably and, particularly in the case of non‐selective bottlenecks, without the accumulation of external signals. It has been proposed that bet‐hedging strategies, such as LH, have evolved in bacteria because these mutational processes generate pre‐selection phenotypic diversity that maximises bottleneck survival.

### 5.1 Contingency loci and the importance of mutability

**Figure 4:**
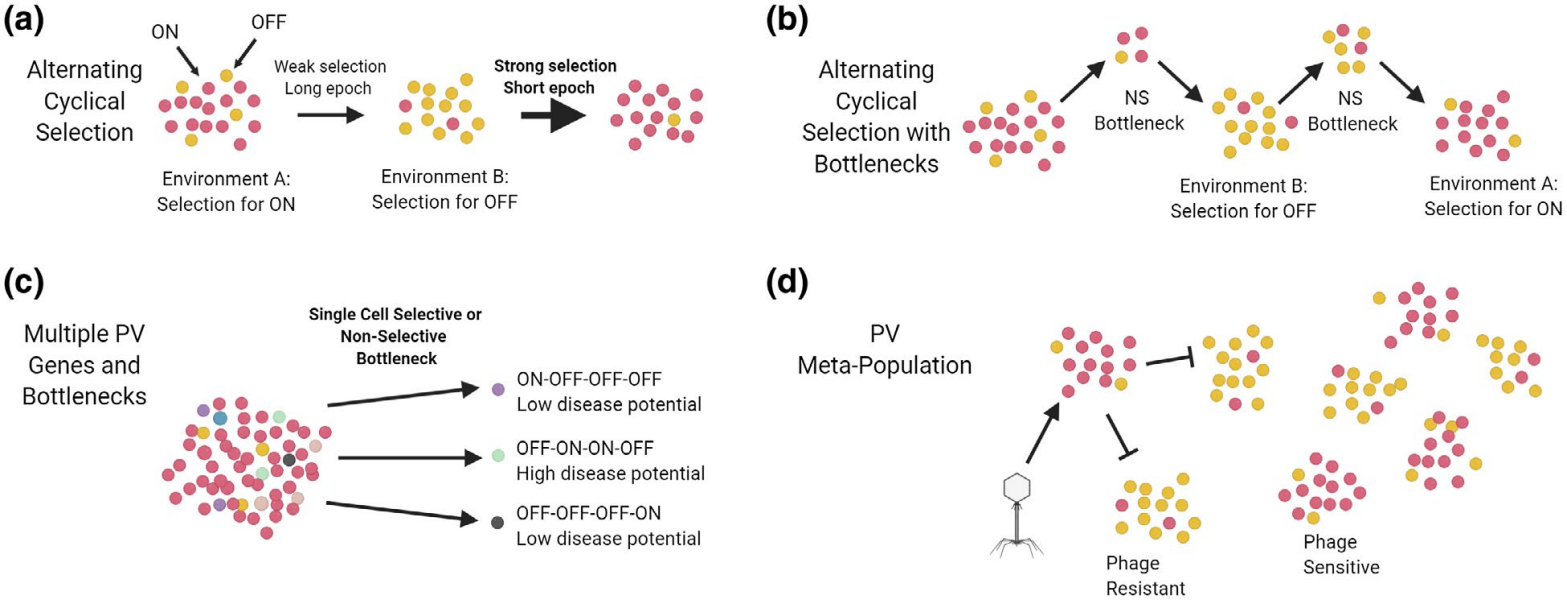
Potential impact of selective and non‐selective bottlenecks on the evolution of LH. In this figure, we depict four scenarios that have been subject to mathematical modelling to understand how selective and non‐selective bottlenecks impact on the evolution of PV and on the potential impact on adaptive outcomes. Panel (a) depicts a simple model where there is alternating selection for the ON and OFF states of a single PV locus. The critical factors in this model are the strength of selection and amount of time spent in each selective environment. Panel (b) depicts a similar model except that a non‐specific bottleneck is imposed between each environment. In this case, population survival depends on both phase variants surviving the bottleneck. Panel (c) depicts how a single cell bottleneck acting on a population containing multiple phase variants (four genes that can switch ON and OFF) can produce diverse outcomes in terms of disease potential. Panel (d) illustrates the new idea of bacterial herd immunity that arises when a meta‐population consists of sub‐populations in different PV states prevents spread of a phage through the whole meta‐population. Image created with BioRender

The principle of LH providing a fitness advantage following exposure to a selective bottleneck was clearly set out by Moxon et al. (1994). They proposed a simple theoretical model to demonstrate how an optimal genomic mutation rate could be achieved by compartmentalising mutability into conserved ‘core’ genes with low rates and contingency loci with substantially higher rates. This theory highlighted how bacterial populations can stabilise occupation of the highest fitness peak in the general landscape while bet‐hedging against rapid exposure to rare but strongly selective environments. There was however no quantification of the advantages of differing switching rates. Extrapolating from observed LH mutation rates, Palmer, Lipsitch, Moxon, and Bayliss (2013) examined the interplay between the number of generations separating each bottleneck event (epoch length) and selection by simulating SSR‐mediated switching between ON and OFF states of a single using locus with either symmetrical or non‐symmetrical selection for each state (Figure 4a). The ‘winning’ mutation rate was the reciprocal of the function of average epoch length and the stringency of the bottleneck, with the outcome of frequent and/or highly selective, bottleneck events favouring higher mutation rates mirroring previous work (Gaál, Pitchford & Wood, 2010; Lachmann & Jablonka, 1996). Contrastingly, Kussell and Leibler (2005) found that responsive environmental sensing is more beneficial than stochastic switching in highly changeable environments, despite a high cost of sensing, whereas stochastic switching was favoured in more constant environments. An interesting observation by Patra and Klumpp (2015) concerned reversible, switching into a slow‐growing persister cell state. This state was beneficial during prolonged periods of stress as the impact of persisters on overall growth rate was mitigated by enhanced survival. Moxon and Kussell (2017) extended this work by modelling the impact of bottlenecks on high frequency phenotypic switching between growing and non‐growing bacteria (Figure 4b). Within this model, non‐growing bacteria maintain a higher transmission probability and, upon transmission, can proliferate whereas there is a reverse effect on the growing cells. A key observation was for an extinction threshold for switching rates below which the PV rate is too low for survival and acts to prevent the SSR from evolving to low repeat numbers. Interestingly, highly stringent selective bottlenecks were required for evolution of LH. These models highlight the potentially crucial role of selective bottlenecks in shaping and maintaining the mutability of LH.

### 5.2 Narrow non‐selective bottlenecks re‐shape LH diversity

Non‐selective bottlenecks represent a contrasting and oblique challenge to a microbial population. The key challenges in this case are how to avoid losing genetic diversity and how to regain diversity in the absence of selection. While LH was well known to produce diversity in the absence selection, recent observations have elaborated some of the operational principles. Several phase‐variable bacterial species can generate high levels of phenotypic diversity through the stochastic, independent ON/OFF switching of multiple phase‐variable loci (Figure 4c). These combinatorial expression states are termed phasotypes and the number of states is a function of the number of loci and expression states. The application of non‐selective bottlenecks to a population acts to reduce phasotypic diversity (i.e. the number and prevalence of phasotypes). Aidley and Colleagues (2017) explored PV of *C. jejuni* strain NCTC11168, which has 28 phase‐variable genes. A randomly selective bottleneck model with no selective inference was applied to populations containing multiple phasotypes. Narrowing of the bottleneck led to development of an increasing bimodal distribution of phasotypes resulting from exclusion of rarer phenotypes from a highly divergent population.

*C. jejuni* is a frequent and natural coloniser of the chicken ceca but also causes a serious gastric infection on humans following ingestion of undercooked meat results (Coker, Isokpehi, Thomas, Amisu & Larry Obi, 2002)*.* Genomes of this species contain multiple PV genes, many of which have known or putative virulence attributes (Bacon et al., 2001; Guerry et al., 2002; Karlyshev, Linton, Gregson & Wren, 2002). Wanford and Colleagues (2018) investigated the dynamics of PV during experimental, asymptomatic colonisation of chickens by a *C. jejuni* strain. Although starting from diverse mix of phasotypes in the inoculum, output populations were dominated by a single phasotype with no evidence for selection as each independent output population was dominated by a different phasotype. Application of the Aidley et al. model to the phasotypes of these experimental *C. jejuni* populations predicted that the low levels of diversity and high levels of divergence were due to a single cell bottleneck during host colonisation (Wanford et al., 2018)*.* It was speculated that these differing phasotypes have dissimilar phenotypes and may alter the propensity for downstream effects on transmission between chickens and on pathology in humans.

These two studies highlight how non‐selective bottlenecks can re‐shape LH‐dependent diversity and introduce the potential for stochastic effects on future selective events as an outcome of a narrow bottleneck is population‐to‐population variation in the dominant phasotype.

### 5.3 Compartmentalisation of PV states

Commonly microbial populations are thought of as randomly mixing homogeneous entities where each member is equally susceptible to change. The inclusion of more compartmentalised conglomerate models can alter the mechanism of bottleneck action. In 2019, Turkington et al. showed that experimental compartmentalisation of stochastic switching states in a meta‐population can act to introduce herd immunity to phage infection (Figure 4d) (Turkington, Morozov, Clokie & Bayliss, 2019). In a similar example, Furi and Colleagues (2019) found that infection of *S. pneumoniae* populations by a phage was dependent on the methylation state of the phage, the number of methylation sites in the phage genome and the predominant phase state of the bacterial SpnIII system that can switch between six differing states. An under‐explored aspect of both of these scenarios was that pre‐exposure to phage or other selective pressures had the potential to influence the meta‐population structure. Thus, understanding the preceding selection history and distribution of PV states in a meta‐population is likely to severely impact on the adaptive potential of LH.

## 6 WHAT PROGRESS HAS BEEN MADE TOWARDS DISCERNING WHETHER LH IS ADAPTIVE FOR OR FACILITATES SURVIVAL OF WITHIN‐HOST OR TRANSMISSION‐ASSOCIATED BOTTLENECKS?

Experimental evidence for the existence of frequent, recurrent and highly narrow and/or stringent bottlenecks during both within‐host spread and transmission between hosts of bacterial organisms is now extensive and nuanced. Conversely the impact of these bottlenecks on genetic diversity, particularly due to LH, is sparse. The key advances we have highlighted are that immunological effectors, cells and organs can be major sources of bottlenecks during bacterial infections and that bottlenecks can be as narrow as a single cell. We have also highlighted the major growth in our understanding of the complexity of LH. The detection of a wide dispersal of both simple and complex PV RM systems and the variable presence of LH within and among species of bacterial genera hints at both a widespread selection for LH (possibly driven by phages) but also niche specialisation limiting the adaptive benefits of hypermutability. Despite these advances, studies of how different types of bottlenecks are impacted by LH are still a rarity and have yet to explicitly demonstrate that LH enables adaptation to, or survival of, both the initial bottleneck and of a subsequent, alternative selective event.

Despite the uncertainties about real‐world bottlenecks and the new findings on LH diversity, mathematical modelling has established a strong basis for unravelling the evolutionary forces acting on LH. The key roles of selection strength, epoch length, bottleneck frequency, bottleneck stringency and compartmentalisation have firm foundations with models that can be applied or adapted to specific situations. There are, of course, still many gaps in our understanding of key processes that mainly stem from a lack of real‐world knowledge; how do we measure selection strength?; are real‐world bottlenecks highly selective for variation in a single genetic locus or multiple loci?; are some bottlenecks completely, or evolutionarily, non‐selective? We also need to be aware of whether specific events are of evolutionary importance or are accidental effects of the existence of certain virulence attributes and LH mechanisms. This latter point is a key concern as theoretical modelling of evolutionary processes needs to focus on attributes that contribute to evolution and not on evolutionary dead ends such as bacterial meningitis. Perhaps unsurprisingly, we still await a definitive theoretically underpinned experimental demonstration that differences in mutability of an LH locus, or even loss of mutability, provide a competitive advantage during multiple cycles of transmission of an infectious agent.

## CONFLICT OF INTEREST

The authors declare no conflicts of interest.

## AUTHOR CONTRIBUTIONS

MDSC, JH, JJW, ERM, MRO and CDB have all made major contributions to the drafting of the manuscript. MDSC, JJW and CDB made and refined figures.
